# Open data phylometabolomics reveals turnover‐dominated chemical divergence and clade‐specific physicochemical regimes across angiosperms

**DOI:** 10.1111/tpj.70820

**Published:** 2026-03-23

**Authors:** Carlos Alexandre Carollo, Amanda Galdi Boaretto, Mariana Calarge Nocetti, Aline Regina Hellmann Carollo, Flávio Macedo Alves

**Affiliations:** ^1^ Laboratory of Natural Products and Mass Spectrometry (LaPNEM) Federal University of Mato Grosso Do Sul Campo Grande MS Brazil; ^2^ LaSMiNano—Laboratory of Micro‐ and Nanostructured Systems Federal University of Mato Grosso Do Sul Campo Grande MS Brazil; ^3^ Laboratory of Botany Federal University of Mato Grosso do Sul (UFMS) Campo Grande MS Brazil

**Keywords:** phylometabolomics, specialized metabolism angiosperms, chemical turnover, beta diversity, Jaccard dissimilarity, macroevolution, chemotaxonomy, physicochemical traits, LOTUS database

## Abstract

Specialized metabolites are central to plant defense, signaling and ecological interactions, yet we still lack a macroevolutionary framework explaining how this diversity is structured across angiosperms. Here, we integrate the open LOTUS chemical repository with standardized taxonomy to map a curated ‘chemical core’ comprising 77 404 family‐supported occurrences across 12 representative families spanning Magnoliids, Monocots, and Eudicots. Chemical composition shows strong higher‐level structure, delineating major lineages while revealing a striking evolutionary topology: inter‐lineage divergence is dominated by metabolite replacement rather than accumulation. Across family pairs, β‐diversity is overwhelmingly explained by turnover, indicating that chemical disparity rarely arises from one lineage retaining subsets of another's repertoire. Despite this universal turnover regime, lineages occupy distinct physicochemical and elemental neighborhoods, consistent with divergent evolutionary strategies under shared allocation constraints. Magnoliids define a lipophilic boundary characterized by greater investment in nitrogen‐bearing and aromatic‐rich defenses; Monocots occupy a more hydrophilic, high‐molecular‐weight and structurally saturated niche; and Eudicots expand oxygen‐rich carbon scaffolds with reduced nitrogen dependence. Together, our results indicate that angiosperm chemical evolution is highly dynamic at the compositional level, yet constrained at the architectural level, with persistent turnover generating lineage‐specific chemical identities within inherited physicochemical corridors. This work provides a reproducible open data foundation for testing mechanistic links between biosynthetic organization, ecological antagonists, and evolutionary diversification of plant chemistry.

## Significance Statement

Using an open, reproducible phylometabolomics pipeline (LOTUS + standardized taxonomy; ~77 000 occurrences), we show that angiosperm chemodiversity diverges primarily by turnover (replacement), not nested accumulation. Despite this dynamism, major clades occupy distinct physicochemical regimes, revealing conserved constraints that channel chemical innovation across deep time.

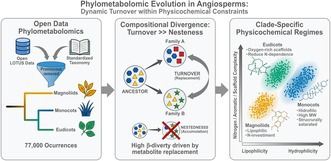

Angiosperm specialized metabolism is often portrayed as cumulative, with derived lineages adding new chemical classes. Using a reproducible framework linking LOTUS occurrences to standardized taxonomy, we show that deep‐lineage chemical divergence is driven by replacement: turnover explains nearly all β‐diversity, while nestedness is minimal; clades occupy distinct physicochemical and elemental regimes that constrain chemical evolution across angiosperms.

## INTRODUCTION

Plant specialized metabolites represent one of the most striking dimensions of biodiversity, underpinning ecological interactions, defense strategies, and adaptive radiations throughout the diversification of flowering plants (Du et al., 2024; Theis & Lerdau, 2003; Wink, 2003). These structurally and functionally diverse molecules, including alkaloids, terpenoids, phenolics, and nitrogen‐containing compounds, mediate key ecological processes such as herbivore deterrence, pollinator attraction, allelopathic interference, and microbial interactions (Adler, 2003; Sánchez‐Moreiras et al., 2003; Vanderplanck et al., 2020). Understanding the diversification of specialized metabolism is therefore fundamental to explaining both the evolutionary success of angiosperms and their continued ecological and societal relevance.

While the genomic basis of metabolic diversity has become increasingly well understood, driven by gene duplication, neofunctionalization, and the assembly of metabolic gene clusters (Chu et al., 2011; Grotewold, 2005; Osbourn, 2010; Smit & Lichman, 2022), the macroevolutionary trajectory of the chemical phenotype itself remains poorly resolved. At large evolutionary scales, a central question persists: does the chemical space of angiosperms expand through the gradual accumulation of traits, or is it shaped by rapid cycles of innovation and loss? Although biochemical pathways such as the phenylpropanoid, terpenoid, and alkaloid networks have been deeply characterized (Cheynier et al., 2013; Ferrer et al., 2008; Yonekura‐Sakakibara et al., 2019), we still lack a quantitative framework to determine how chemical diversity is structured across plant families and orders.

Historically, phytochemical research has relied on lineage‐focused analyses, such as alkaloids in Amaryllidaceae (Desgagné‐Penix, 2021; He et al., 2015; Jin & Yao, 2019), phenolics in Poaceae (Fatima et al., 2021; Míka et al., 2005), terpenoids in Asteraceae (Proksch & Rodriguez, 1983; Wu et al., 2006), or lignans and isoquinoline alkaloids in Lauraceae (Custódio & Veiga‐Junior, 2014; Li et al., 2018). These studies have illuminated biosynthetic mechanisms and ecological functions but provide only a fragmented view of chemical diversification.

The lack of harmonized, large‐scale chemical datasets has hindered robust comparative analyses. Traditional bibliographic repositories such as SciFinder and Web of Science, while extensive, proved inadequate for comprehensive phylogenetic mapping due to structural biases toward non‐specialized metabolites and restrictive data access policies. Moreover, the absence of persistent identifiers linking molecular structures to taxonomic provenance in these proprietary databases prevents the large‐scale verification required for evolutionary studies. Consequently, macroevolutionary hypotheses have frequently relied on generalized assumptions about ‘advanced’ versus ‘basal’ chemistry without empirical phylogenetic grounding.

This limitation has begun to shift with the emergence of open, FAIR‐compliant natural product repositories, exemplified by the LOTUS initiative (Rutz et al., 2022; Wilkinson et al., 2016). By aggregating chemical structures, biological sources, and metadata under standardized principles, such resources enable cross‐lineage comparisons at an unprecedented scale. When paired with globally curated taxonomic frameworks such as World Flora Online (WFO, 2025), they allow the disentanglement of genuine evolutionary patterns from the pervasive sampling biases present in the phytochemical literature (Petrén et al., 2024; Pichersky & Lewinsohn, 2011; Ramos et al., 2023; Wink, 2008). This convergence of chemical informatics and phylogenetics makes it possible to transition from traditional chemotaxonomy to a data‐driven ‘phylometabolomics’.

Taxonomic structuring of plant chemistry can arise through multiple evolutionary processes. Conserved biosynthetic pathways may promote similarity among related taxa, whereas convergent evolution can generate chemically analogous metabolites in phylogenetically distinct lineages. Angiosperm families exemplify a broad range of contrasting chemical strategies, from nitrogen‐rich alkaloids to polyphenolic defenses and volatile terpenoids. Yet whether these diverse traits reflect conserved metabolic cores or rapidly shifting chemical identities remains unknown (Pichersky & Lewinsohn, 2011; Pichersky & Raguso, 2018; Wink, 2003).

A quantitative understanding of chemical diversification requires integrating statistical ecology, cheminformatics, and macroevolutionary theory. Diversity metrics, including richness, effective diversity, and β‐diversity partitioning, enable total chemical variability to be decomposed into turnover and nestedness components, distinguishing whether diversification arises through the accumulation or replacement of metabolites (Baselga, 2010; Petrén et al., 2024). Complementarily, the concept of chemical space provides a multidimensional representation of the structural and physicochemical landscape of metabolites (Domingo‐Fernández et al., 2023), while descriptors such as molecular weight, polarity, and lipophilicity help link metabolic architecture to biosynthetic constraints and ecological trade‐offs (Chu et al., 2011; Smit & Lichman, 2022).

In this study, we integrate LOTUS chemical data with the World Flora Online taxonomy to analyze 77 404 high‐confidence occurrences across 12 representative angiosperm families spanning Magnoliids, Monocots, and Eudicots. Through an open and fully reproducible R‐based workflow, we test two macroevolutionary models of metabolic diversification: (1) an Accumulation Model, where derived lineages retain ancestral chemistry while adding new complexity; and (2) a Turnover Model, where chemical repertoires are frequently replaced through rapid innovation and loss. Furthermore, we investigate whether these shifts follow directional physicochemical trajectories, specifically toward increased polarity and structural complexity, assessing how trade‐offs and constraints on molecular weight, polarity, and nitrogen investment shape metabolic evolution across major angiosperm clades. By bridging chemical informatics, evolution, and ecology, this framework establishes a quantitative basis for understanding how plant chemical diversity is generated, maintained, and reshaped across deep evolutionary time.

## RESULTS AND DISCUSSION

### An integrated framework for Phylometabolomics

**Figure 1:**
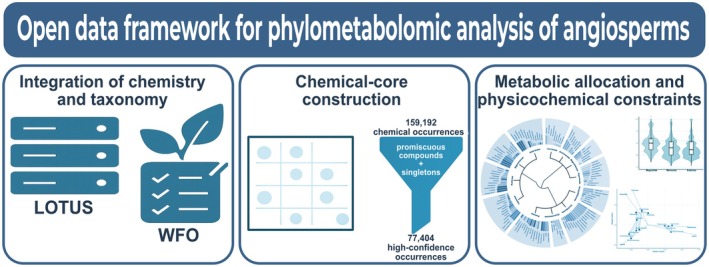
Open data framework for phylometabolomic analysis of angiosperms. The workflow integrates open chemical data (LOTUS) with global taxonomic standards (World Flora Online, WFO) to harmonize raw chemical occurrences and generate a curated ‘chemical core’ by removing highly promiscuous compounds and within‐family singletons. This core matrix underpins downstream analyses of chemical diversity, β‐diversity, multivariate ordination, and family class networks, revealing the macroevolutionary structure and physicochemical divergence of specialized metabolism across angiosperm lineages.

To overcome the fragmentation of chemical data across disparate sources, we developed a fully reproducible phylometabolomic framework that integrates open chemical repositories with standardized taxonomy (Figure 1). The pipeline begins by harvesting raw chemical occurrences from the LOTUS database and harmonizing all plant names against the World Flora Online backbone to resolve taxonomic synonyms and ambiguities. The core of the framework is a rigorous filtering stage designed to separate lineage‐specific metabolic traits from ubiquitous or promiscuous compounds. By removing these widespread entities and unstable singleton records, the workflow yields a curated ‘chemical core’ high‐confidence occurrences suitable for macroevolutionary analysis. This high‐confidence matrix feeds a multi‐tier analytical suite, including chemotaxonomic clustering, diversity partitioning, multivariate ordination, and network analysis, to map how specialized metabolism diversifies across the angiosperm tree of life. This structured approach ensures that the resulting patterns reflect genuine biological signals rather than sampling artifacts or data heterogeneity.

### Filtering effects and data refinement

Sequential filtering produced a refined and ecologically interpretable core for evolutionary comparisons across the 12 focal families. From 159 192 LOTUS species–compound occurrences, we applied a two‐step procedure to separate lineage‐structured specialized metabolism from broadly shared background chemistry. First, a promiscuity filter removed compounds recorded in ≥6 families or present in ≥20% of species, which otherwise inflate apparent similarity among distant clades and obscure chemotaxonomic signal. Second, we required within‐family support (occurrence in ≥2 species per family) to minimize singletons that are disproportionately sensitive to sampling artifacts and residual taxonomic uncertainty.

This curation retained 77 404 high‐confidence occurrences (48.6%), defining a ‘chemical core’ enriched in recurrent, family supported chemotypes (Table 1). Notably, retention differed sharply among lineages: families dominated by lineage‐restricted pathways (e.g., Amaryllidaceae and Piperaceae) retained a larger share of their reported chemistry, whereas others (e.g., Bignoniaceae and Magnoliaceae) showed steeper attrition, indicating that a larger fraction of their currently described metabolomes is composed of widely distributed or weakly supported metabolites.

**Table 1:** Chemical diversity, sampling coverage, and retention of lineage‐specific metabolites across 12 angiosperm families

Clade	Family	Initial compounds^a^	Core compounds (retained)^b^	Retention (%)^c^	Initial species	Kept species
Monocots	Amaryllidaceae	2011	532	26.5	211	204
	Dioscoreaceae	790	131	16.6	55	47
	Orchidaceae	2105	358	17.0	218	174
	Poaceae	2000	351	17.6	186	159
Magnoliids	Annonaceae	4109	760	18.5	306	258
	Lauraceae	3385	653	19.3	268	227
	Magnoliaceae	861	134	15.6	48	44
	Piperaceae	2036	536	26.3	125	108
Eudicots	Asteraceae	34 531	8743	25.3	3787	3435
	Bignoniaceae	1267	189	14.9	92	74
	Fabaceae	16 015	3888	24.3	2244	2032
	Rutaceae	7051	1692	24.0	538	501

^a^ Initial compounds: Total unique structures (InChIKeys) retrieved from LOTUS.

^b^ Core compounds: Metabolites retained after excluding promiscuous compounds (occurring in >6 families or present in ≥20% of all species) and singleton records (occurring in only 1 species per family).

^c^ Retention: Percentage of the original dataset comprising lineage‐specific core metabolites.

The 353 excluded promiscuous metabolites were pervasive: By querying LOTUS for each metabolite's recorded taxonomic breadth (i.e., the number of distinct families and species associated with that compound), we found that they occurred in 60.0 ± 44.6 families and 253.7 ± 308.6 species on average, with extreme cases spanning up to 325 families and >2900 species (Table S1). Rather than being uninformative ‘noise’, this ubiquity is consistent with a diffuse metabolic background generated by enzymes with relaxed specificity, where low‐abundance side‐products and structurally adjacent analogs are continuously produced and intermittently stabilized under selection (Kreis & Munkert, 2019; Leong & Last, 2017).

In plants, the catalytic breadth of large enzyme superfamilies, particularly cytochrome P450 monooxygenases, provides a plausible mechanistic substrate for this phenomenon by enabling repeated oxidative tailoring across multiple scaffolds and, consequently, the widespread recurrence of certain phenolic and terpenoid motifs across lineages (Werck‐Reichhart, 2023). Within this framework, filtering does not discard biological relevance; it explicitly separates evolutionary layers: a broadly shared ‘background’ chemistry expected under pervasive enzymatic promiscuity, and a lineage‐supported layer that is more likely to represent conserved biosynthetic modules or stabilized lineage‐specific adaptations.

Beyond absolute richness, normalizing core compound counts by species diversity revealed contrasting diversification among families. Piperaceae displayed the highest chemical density (≈4.96 compounds/species), consistent with a strategy of high biosynthetic elaboration per lineage in which diversification arises through extensive derivatization within species, an interpretation aligned with the documented propensity of Piperaceae to generate large suites of structurally related neolignans, amides, and chromene‐like scaffolds from a limited set of biosynthetic entry points (Kato & Furlan, 2007). In contrast, Fabaceae, despite massive total richness, showed lower density (≈1.91 compounds/species), implying that its chemical diversification is driven more by extensive lineage radiation than by unusually large per‐species repertoires. This pattern is compatible with the view that many legume defenses are dynamically recruited and inducible (e.g., isoflavonoid‐centered chemotypes), potentially reducing the selective premium on high constitutive chemical loads while enabling rapid ecological specialization across a large species pool (Wink, 2013).

At the clade scale, Magnoliids exhibited the highest average chemical density (3.46), exceeding Monocots (2.41) and Eudicots (2.60), consistent with the hypothesis that Magnoliids rely on chemically complex, often locally deployed defenses (Gottlieb, 1990; Meldau et al., 2012).

**Figure 2:**
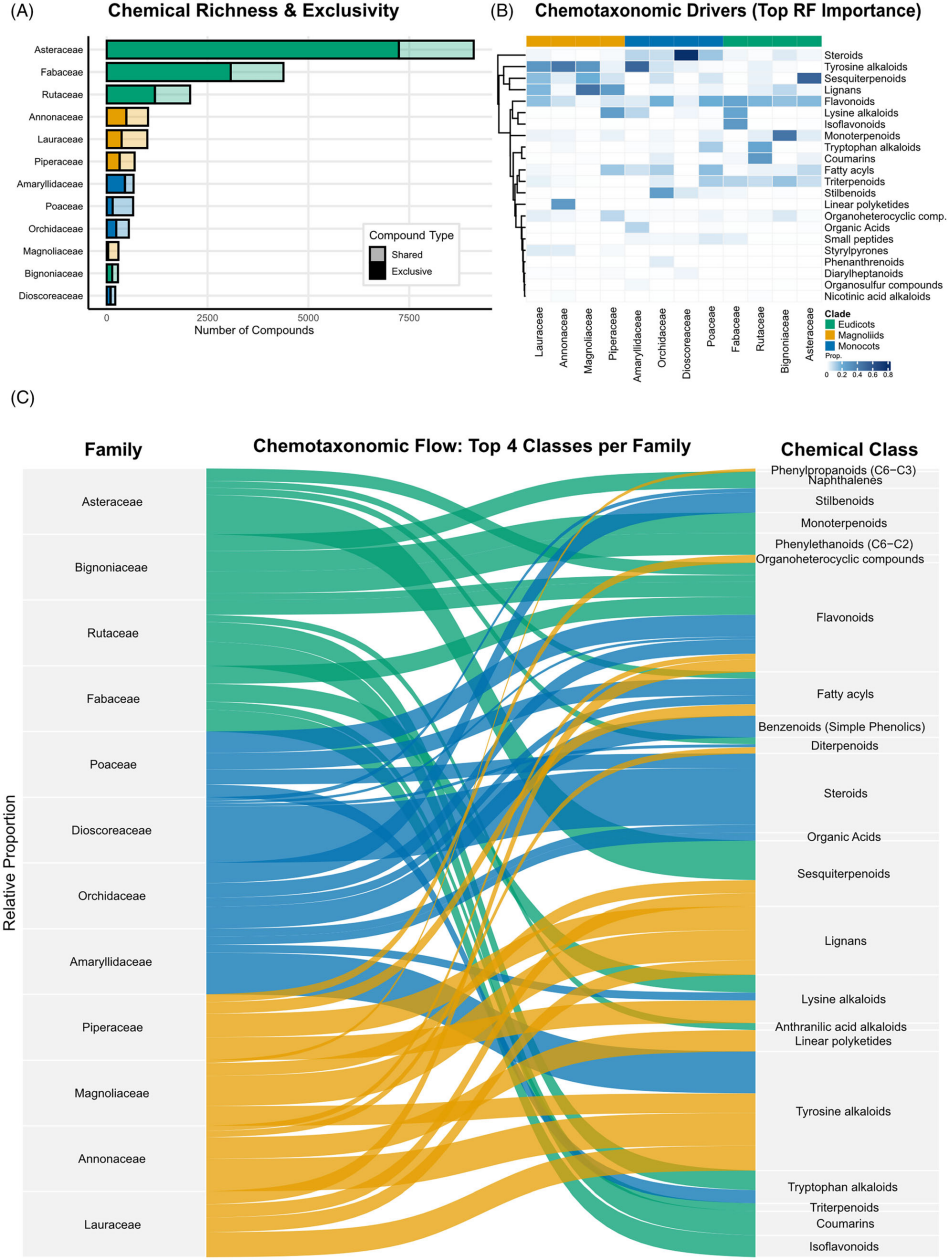
Integrated structure of chemical diversity across angiosperm lineages. (A) Richness and turnover: dark segments represent family exclusive compounds. (B) Phylogenetic chemical fingerprints: heatmap of the top Random Forest chemical drivers. (C) Biosynthetic flow: alluvial diagram linking families to their major biosynthetic classes.

Finally, only 8.5% of the core chemical diversity is shared across families, as the cumulative sum of family‐specific richness (17 967) exceeded the global unique count (16 436) by just 1531 metabolites. Rather than mere redundancy, this limited overlap represents the ‘connective tissue’ of angiosperm specialized metabolism: evidence for the recurrent recruitment of ancestral biosynthetic modules and the convergent stabilization of chemical solutions under comparable ecological pressures. Critically, the within‐family support filter achieved this increase in evolutionary interpretability without major taxonomic erosion, as species retention remained high (~90%) across lineages (Figure 2A). Together, these results show that a large fraction of reported phytochemistry indeed reflects a widespread background expected under enzymatic promiscuity, but that a substantial, family supported core persists and can be meaningfully interpreted as stabilized evolutionary signal, setting the stage for testing whether diversification across clades is driven primarily by accumulation or by extreme lineage‐specific turnover.

### Chemotaxonomic signatures and biosynthetic allocation across angiosperms

The chemotaxonomic structure underlying these patterns becomes clearer when examining the Random Forest chemical drivers (Figure 2B). The heatmap reveals a strongly partitioned chemical landscape in which families differ not only in the presence of specific classes but, more importantly, in the relative intensity of their biosynthetic investments.

Among Magnoliids, this specialization forms a coordinated block of aromatic scaffolds: Lauraceae and Annonaceae are enriched in tyrosine‐derived alkaloids (30 and 44%, respectively), while Magnoliaceae and Piperaceae exhibit a pronounced lignan signal (47 and 28%), with additional alkaloid enrichment in Piperaceae (lysine alkaloids, 27%). Collectively, these profiles define a cohesive Magnoliid signature centered on nitrogen‐expensive chemistry paired with structurally conserved phenylpropanoid‐derived defenses.

This pattern contrasts sharply with eudicot families, which share a broad phenolic foundation but diverge through strong family specific axes combining flavonoids with distinct terpenoid or aromatic modules. Bignoniaceae displays a unique signature driven by monoterpenoid‐derived chemistry (iridoids), which constitutes 46% of its profile, alongside a substantial investment in triterpenoids (14%). This is consistent with the iridoid pathway acting as a key chemotaxonomic marker within Lamiales (Frezza et al., 2019; Jensen et al., 2002). The signal for Fabaceae is equally striking: isoflavonoids emerge as its strongest discriminatory feature (29%), forming an almost lineage‐exclusive chemical axis that separates this family from all others, while Asteraceae allocates heavily to sesquiterpenoids (53%) and flavonoids (17%), in line with classic chemotaxonomic evidence that structurally diverse sesquiterpene lactones represent hallmark metabolites of the family (Seaman, 1982). Rutaceae is defined by an alternative aromatic axis (coumarins, 29%) combined with tryptophan alkaloids (24%), further emphasizing that eudicot differentiation is organized as a shared flavonoid backbone with divergent lineage‐specific elaborations.

Monocots, positioned between these extremes, display heterogeneity that is informative about evolutionary strategy. Dioscoreaceae provides the most extreme example of canalization in the dataset, funneling 79% of its diagnostic chemistry into the steroid pathway, consistent with a lineage specialized in steroidal saponins and related scaffolds. In contrast, Orchidaceae is characterized by lineage‐specific stilbenoids (27%, nearly restricted to this group within our dataset) together with flavonoids (19%) (Veerraju et al., 1989), whereas Poaceae maintains a more balanced portfolio supported by flavonoids (20%), fatty acyls (16%), and steroids (14%). Amaryllidaceae, in turn, shows strong enrichment in its characteristic alkaloid class (53%), underscoring a distinct monocot chemical axis separate from both magnoliid aromatic–nitrogen profiles and Eudicot phenolic–terpenoid elaborations.

These chemical identities are further clarified in the biosynthetic flow diagram (Figure 2C), which translates the statistical drivers into an explicit map of metabolic allocation. The alluvial representation shows that each family channels metabolic resources into a distinctive but internally coherent set of pathways, spanning a continuum from strong canalization (e.g., Dioscoreaceae → steroids) to more distributed investment across multiple modules (e.g., Asteraceae and Poaceae). At the clade scale, these family signatures resolve coherent threads of chemical organization: Magnoliids are anchored by tyrosine‐derived alkaloids (27%) and lignans (23%), monocots are distinguished by a strong steroid signal (28%) largely driven by Dioscoreaceae but supplemented by family‐specific phenolic modules (e.g., stilbenoids in Orchidaceae; flavonoids/fatty acyls in Poaceae), and eudicots share a flavonoid backbone (18%) while clade‐level diversification is expressed through lineage‐specific elaborations (sesquiterpenoids in Asteraceae, coumarins in Rutaceae, and diagnostic isoflavonoids in Fabaceae).

Together, these patterns illustrate how the macroevolution of plant chemistry integrates both diversification and constraint: While lineages replace most of their metabolites through extreme turnover, they do so within the boundaries of clade‐specific resource allocation strategies that shape the recurrent chemical synapomorphies observed across the angiosperm tree.

### Chemotaxonomic architecture of angiosperm chemical space

**Figure 3:**
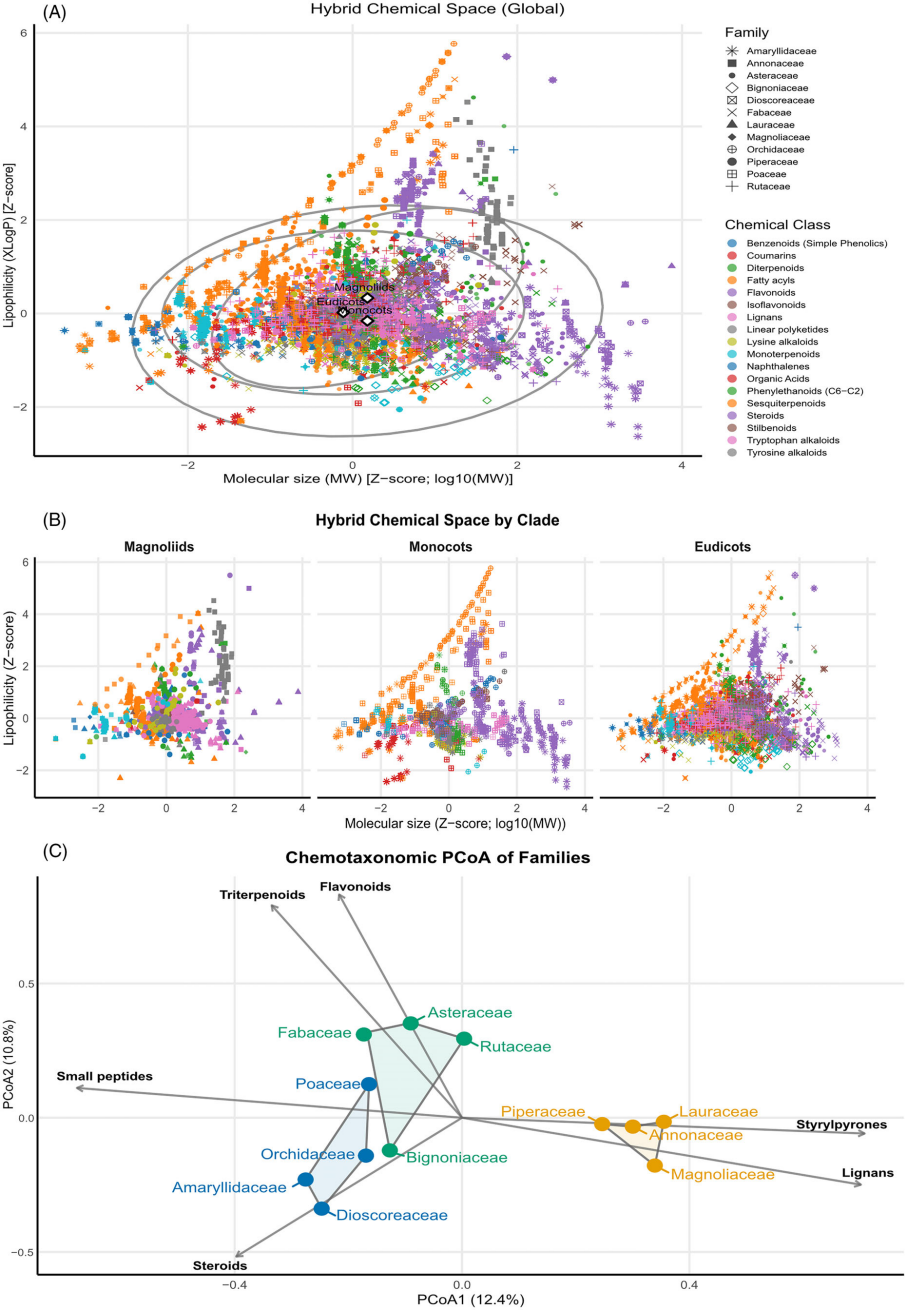
Global hybrid chemical space and chemotaxonomic drivers across angiosperm lineages. (a) Hybrid chemical space defined by standardized molecular size (log10(MW), *Z*‐score) and lipophilicity (XLogP, *Z*‐score). (b) The same chemical space shown separately for Magnoliids, Monocots, and Eudicots. (c) Chemotaxonomic PCoA of families based on Jaccard dissimilarity in chemical composition (PERMANOVA: *R* ^2^ = 0.22, *P* = 0.001).

In the global hybrid map defined by standardized molecular size (MW) and lipophilicity (XLogP), metabolites form a continuous but clearly structured cloud in which increases in size tend to co‐vary with higher lipophilicity (Figure 3A). The low‐MW, hydrophilic edge is enriched in small, polar chemistries (e.g., organic acids and simple phenolics), whereas the upper‐right extremes are populated by bulkier hydrophobic scaffolds typical of terpenoid‐ and lipid‐like architectures. When this landscape is partitioned by clade (Figure 3B), the phylogenetic signal becomes visually explicit: Magnoliids occupy a relatively compact region with selective excursions toward lipophilic space, Monocots show a strongly anisotropic distribution shaped by lineage‐specific corridors, as the observed for the fatty acids, and Eudicots span the broadest area yet retain internal structure rather than a diffuse spread. This pattern supports a key inference for angiosperm chemical evolution: While individual compounds are frequently replaced, the physicochemical boundaries that each lineage tends to occupy behave as conserved traits, plausibly reflecting persistent biosynthetic capacity and organismal constraints on storage, transport, and deployment of specialized metabolites (e.g., secretory tissues, resin/oil systems, vacuolar handling) (Weng et al., 2021).

Family level panels reinforce that clade‐level footprints are assembled from distinct strategies of physicochemical niche occupation (Figure 3B; Figure S2). Three families illustrate the extremes particularly well. Annonaceae shows a bimodal architecture, combining a dense central core with a pronounced excursion into more lipophilic/high‐molecular sectors, consistent with a lineage that repeatedly draws on strongly membrane‐permeable or storage‐compatible scaffolds alongside more polar components, an arrangement expected for defenses deployed through tissue‐localized sequestration and rapid‐release syndromes (Aminimoghadamfarouj et al., 2011).

Dioscoreaceae, by contrast, exhibits one of the most canalized footprints, organized along a narrow high‐MW corridor with relatively constrained dispersion in XLogP, consistent with a steroid‐/saponin‐centered chemical program in which diversification occurs largely within a tightly bounded structural regime rather than by broad exploration of chemical space (Sautour et al., 2007).

At the opposite end, Asteraceae displays a wide, densely populated footprint with strong coverage across MW and XLogP, indicating a large, heterogeneous chemical repertoire consistent with a broad‐spectrum defense portfolio; in our dataset, this expansion is dominated by sesquiterpenoids (53.3%) and flavonoids (16.8%), aligning with the prominence of sesquiterpene lactone‐like scaffolds in this family (Seaman, 1982). To summarize family level class composition, we provide a global overview of chemical‐class repertoires across the 12 focal families (Figure S3).

Together, these observations reconcile rapid compositional turnover with stable chemotaxonomic structure: families may repeatedly replace the specific metabolites they deploy, but diversification remains channeled by inherited constraints that keep each lineage within characteristic physicochemical and biosynthetic neighborhoods.

### Phylogenetic constraints define divergent physicochemical landscapes

**Figure 4:**
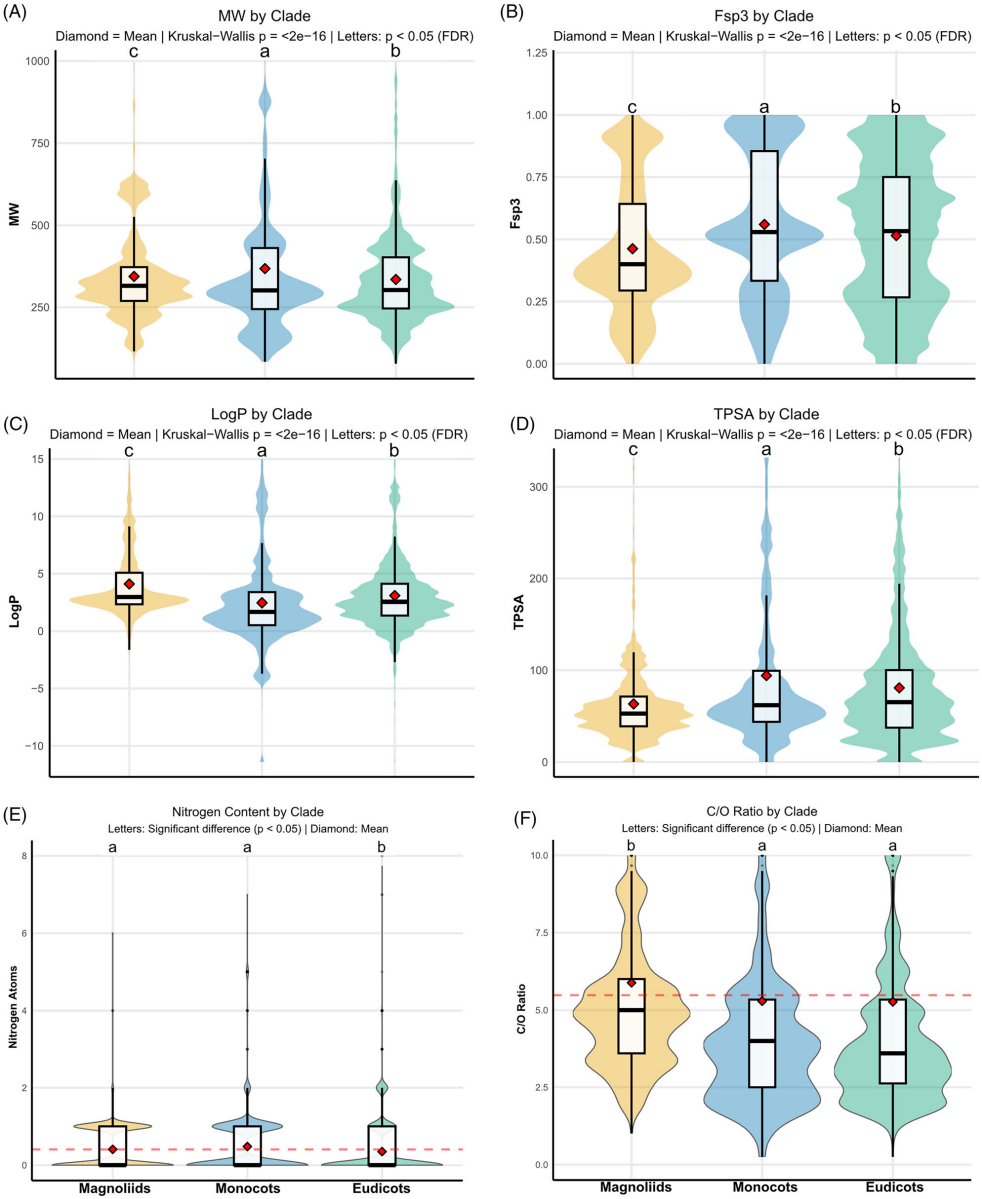
Physicochemical and elemental divergence among major angiosperm clades. (A–F) Violin plots summarize the distributions of six molecular descriptors for Magnoliids (green), Monocots (yellow), and Eudicots (blue): (A) molecular weight (MW); (B) fraction of sp^3^ carbons (Fsp^3^); (C) lipophilicity (XLogP); (D) topological polar surface area (TPSA); (E) nitrogen atom counts per metabolite; (F) carbon‐to‐oxygen ratio (C/O). Diamonds indicate the mean and central bars the median. Letters denote statistically significant differences among clades (Kruskal–Wallis *P* < 2 × 10^−16^; pairwise FDR‐corrected *P* < 0.05).

Phylogenetic lineages occupy statistically distinct regions of physicochemical space (Figure 4), indicating that metabolite replacement is coupled to directional shifts in scaffold architecture and elemental investment rather than undirected drift. Magnoliids (Lauraceae, Annonaceae, Magnoliaceae, Piperaceae) define the most lipophilic metabolome, with significantly higher XLogP (4.10 ± 2.91) and lower TPSA (63.18 ± 47.76 Å^2^) than Monocots (2.47 ± 3.82; 94.13 ± 88.50 Å^2^) and Eudicots (3.09 ± 2.98; 80.76 ± 65.97 Å^2^) (Kruskal–Wallis, *P* < 0.001; FDR: Magnoliids > Eudicots > Monocots for XLogP).

This physicochemical bias is mirrored by scaffold saturation: Magnoliids show the lowest Fsp^3^ (0.462 ± 0.263), whereas Monocots are the most saturated/three‐dimensional (0.560 ± 0.289) and Eudicots are intermediate (0.515 ± 0.288) (Kruskal–Wallis, *P* < 0.001). Monocots simultaneously exhibit the highest mean molecular weight (367.91 ± 218.01 Da), exceeding Magnoliids (344.00 ± 135.95 Da) and Eudicots (334.76 ± 146.04 Da) (*P* < 0.001), supporting a ‘hydrophilic–complex’ niche compatible with bulky, highly functionalized, and often glycosylated chemistries.

In contrast, Eudicots are distinguished less by extreme size than by oxidative expansion and nitrogen economy. Both Eudicots and Monocots show reduced C/O ratios relative to Magnoliids (Eudicots 5.27; Monocots 5.29 vs. Magnoliids 5.88). Most notably, Eudicots contain significantly fewer nitrogen atoms per metabolite (0.26 ± 0.63) compared to the nitrogen‐rich metabolomes of Magnoliids (0.41 ± 0.55) and Monocots (0.51 ± 0.75) (*P* < 0.001; FDR: Eudicots < Magnoliids < Monocots). Together, these results support a macroevolutionary trade‐off in which derived lineages expand chemical diversity through oxygen‐rich carbon scaffolds (phenylpropanoids/flavonoids) while reducing reliance on nitrogen‐expensive alkaloid strategies, an allocation shift expected to be advantageous when nitrogen constrains growth (Ohnmeiss & Baldwin, 1994).

Importantly, this physicochemical divergence can be interpreted as the chemical counterpart of the reproductive transition across angiosperms. Many Magnoliid lineages rely strongly on olfactory attraction and ‘mess‐and‐soil’ pollination modes (notably cantharophily), where visitors often consume pollen and floral tissues (Endress, 2010; Thien et al., 2000). In this ecological setting, a lineage‐wide bias toward low‐TPSA, lipophilic, aromatic‐rich scaffolds is consistent with enhanced partitioning into volatile oils and the deployment of membrane‐permeable defenses. The absence of a nitrogen ‘discount’ in Magnoliids relative to Eudicots is also compatible with stronger constitutive investment in N‐bearing compounds (alkaloids) to protect high‐value reproductive tissues under destructive visitation regimes.

By contrast, the Eudicot radiation is tightly linked to the diversification of floral signaling and visually mediated attraction, where water‐soluble phenolics contribute pigments and optical cues alongside nectar‐mediated rewards (Bachelier & Fay, 2020; Endress, 2010). In this framework, our ‘Nitrogen Economy’ signal provides a mechanistic bridge between reproductive ecology and chemical evolution: by reallocating defense and signaling toward oxygenated, carbon‐based pathways while sparing nitrogen for primary metabolism, Eudicots likely increased metabolic output and ecological flexibility, reinforcing the shift from scent/tissue‐backed rewards to renewable, carbohydrate‐based reward systems.

### Universal constraints: red queen dynamics, turnover pulses, and chemical democracy

**Figure 5:**
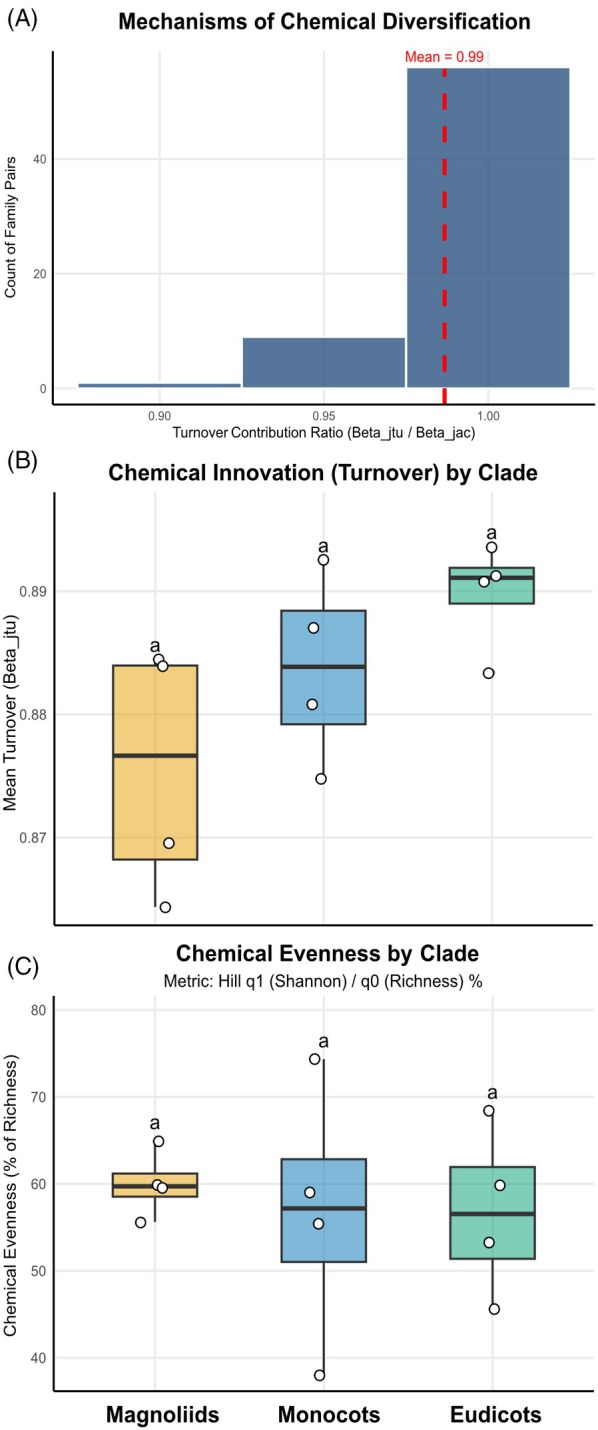
Universal constraints on angiosperm metabolic diversity. (A) The mechanism of diversification: histogram of the turnover ratio. (B) Conserved turnover rates: comparison of turnover values across major clades. The lack of significant differences (Kruskal–Wallis, *P* = 0.146). The lowercase letters indicate statistical groupings from post hoc multiple‐comparison tests. (C) Chemical democracy: analysis of chemical evenness (Hill ratio q1/q0, *P* = 0.694). The lowercase letters indicate statistical groupings from post hoc multiple‐comparison tests.

A central result of our macrochemical comparison is that compositional differences among angiosperm families are overwhelmingly explained by replacement rather than accumulation. Across all 66 family pairs, the turnover component accounts for 98.7% of total Jaccard β‐diversity on average (median 99.4%), leaving only ~1.3% attributable to nestedness (Figure 5A). This means that, at the scale of deep lineages, chemical disparity is rarely generated by one lineage behaving as a subset of another; instead, families diverge primarily by substituting large fractions of their metabolite repertoires.

At the microevolutionary scale, within‐species chemotype diversity can be shaped by genetic architecture together with geography, environment, and demography (Katz et al., 2021). This provides a complementary framework for understanding how standing chemotypic variation and convergence may scale to persistent among‐lineage divergence through lineage sorting and ecological filtering over deep time.

Conceptually, this pattern matches the macroevolutionary logic of ‘taxon–taxon replacement’, long‐term persistence is achieved not by retaining ancestral solutions, but by the continuous reshuffling of functional portfolios under strong ecological coupling. Importantly, the magnitude of turnover itself is high and statistically homogeneous across major clades: mean β_JTU (Jaccard Turnover Component) spans a narrow range (0.86–0.89), and clade‐level comparisons show no significant difference (Kruskal–Wallis, *P* = 0.146) (Figure 5B). This invariance is informative because evolutionary theory frames long‐term change as a balance between a Red Queen steady‐state, dominated by biotic interactions, and a Stationary regime, where change is concentrated in bursts driven by abiotic perturbations (Liow et al., 2011; Stenseth & Smith, 1984). Our data are most parsimoniously consistent with a Red Queen‐like baseline at the level of chemical traits—a persistent pressure for innovation that does not relax even in older lineages like the Magnoliids. While the dominance of turnover is theoretically compatible with the ‘burst‐and‐reset’ dynamics of the Turnover Pulse Hypothesis (Vrba, 1993), the striking uniformity of our results across clades of vastly different ages implies that biotic drivers (e.g., herbivory) likely provide the continuous, universal impetus for chemical replacement, potentially punctuated by abiotic events.

Mechanistically, this replacement‐dominated regime aligns with classic plant–enemy coevolutionary logic. Under the ‘Escape and Radiate’ hypothesis (Ehrlich & Raven, 1964), defensive metabolites function as transient solutions: once antagonists adapt to a specific chemical defense, selection shifts toward novel scaffolds to regain efficacy. Our results provide quantitative support for this model: phylogenetic divergence is not accompanied by nestedness, as would be expected under accumulation, but by repeated loss–gain cycles that continuously remodel the lineage's chemical phenotype.

Finally, the constancy of Chemical Evenness across clades (Kruskal–Wallis, *H* = 0.731, *P* = 0.694) (Figure 5C) suggests an orthogonal constraint on metabolic organization. Even when lineages differ dramatically in species richness, their metabolomes exhibit a conserved rank–abundance topology: A small set of dominant chemical metabolites supported by a long tail of rare analogs. This chemical democracy likely reflects a macroecological zero‐sum constraint on available energy. Metabolic flux can be shifted among pathways but cannot be indefinitely increased without trade‐offs.

These framing bridges ecology and macroevolution by suggesting that energetic constraints impose a stable ceiling on metabolic output, forcing divergent clades to converge on similar evenness profiles even as they diverge sharply in composition via replacement. This decoupling of chemical turnover from lineage diversification rates is particularly evident in the Magnoliidae. Although this clade exhibits heterogeneous and often slower net diversification compared to rapidly radiating Eudicots, its chemical turnover remains consistently high. This indicates that the ecological imperative to innovate metabolically is a constant survival pressure that persists regardless of the tempo of species proliferation.

### Turnover‐dominated divergence and lineage strategies: plasticity versus canalization

Collectively, the results above point to a shared macroevolutionary regime in which angiosperm chemical divergence is persistently turnover‐dominated. This is consistent with Red Queen dynamics (Liow et al., 2011), yet also compatible with Stationary scenarios where episodic abiotic perturbations modulate the tempo of change (Stenseth & Smith, 1984).

**Figure 6:**
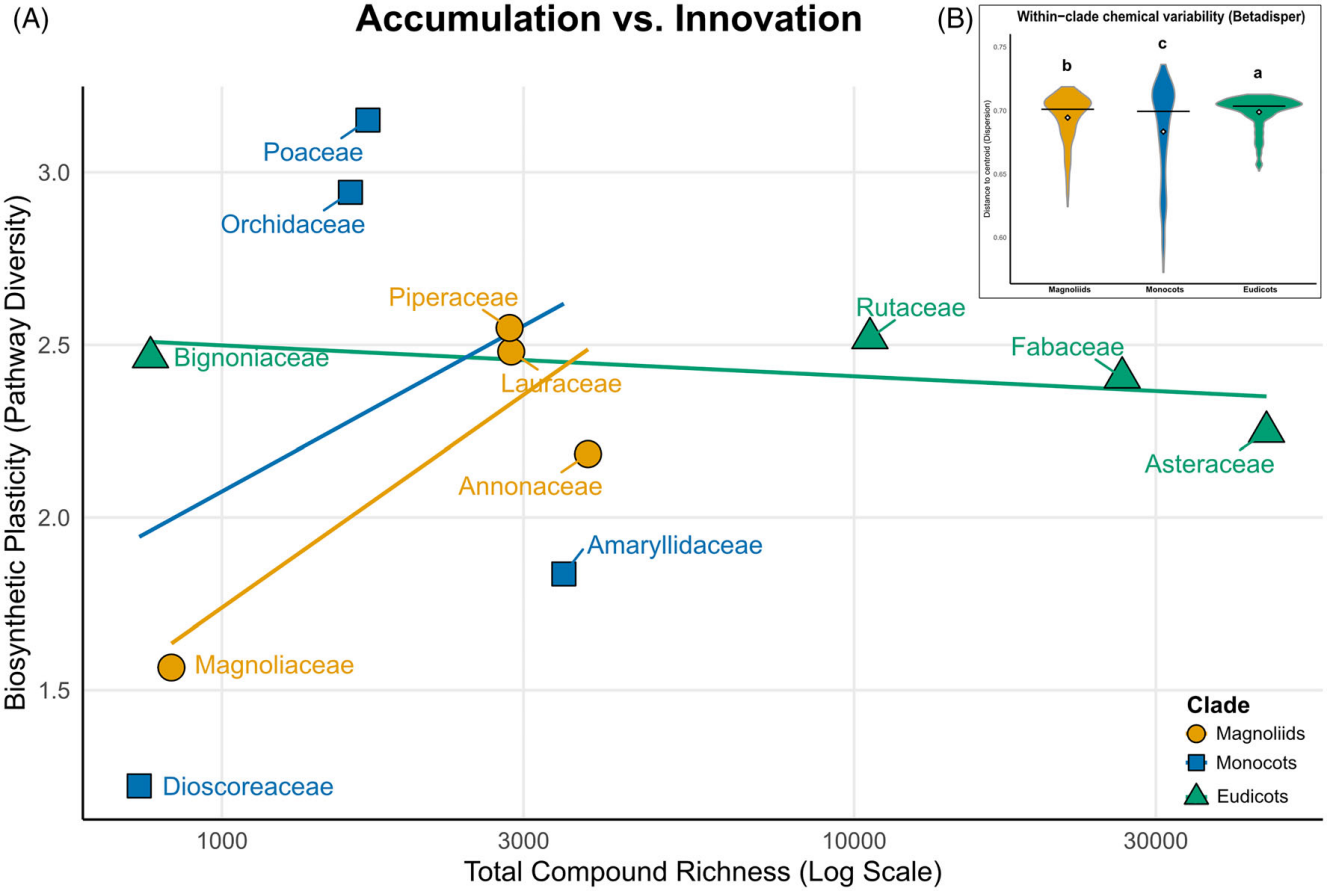
Divergent evolutionary strategies in chemical space exploration. (A) Plasticity versus richness trade‐off: scatter plot correlating total compound richness (X‐axis, Log scale) with biosynthetic plasticity (Y‐axis, Shannon diversity of chemical classes) (Pearson's *R* = 0.20, *P* = 0.536). (B) Expansion of chemical niche: multivariate dispersion (distance to centroid) by clade. Eudicots exhibit significantly higher dispersion than Magnoliids (*P* < 0.001). The lowercase letters indicate statistical groupings from post hoc multiple‐comparison tests.

Within this universal turnover regime, however, lineages differ in the mode by which chemical space is explored. Relating total compound richness (log‐scale) to a Biosynthetic Plasticity Index (Shannon diversity of chemical classes) reveals contrasting positions along an accumulation–innovation continuum (Figure 6A). Families with expansive repertoires but only moderate plasticity (e.g., highly compound‐rich eudicot lineages) are consistent with diversification via expansion within modular frameworks (deepening within dominant biosynthetic domains), whereas families with high plasticity at modest richness reflect broader pathway‐level breadth (Figure 6A). Mechanistically, modular biosynthetic systems, particularly terpenoid metabolism, provide a plausible substrate for such diversification because chemically diversified portfolios can enhance defense against suites of enemies and may hinder resistance evolution (Pichersky & Raguso, 2018).

These lineage‐level strategies are compatible with a biotically structured selective landscape in which divergence in defensive traits promotes herbivore specialization, which in turn feeds back to maintain chemical dissimilarity among plant lineages and within communities (Becerra, 2015). Consistent with this view, within‐lineage chemical variability differs among clades (betadisper; permutational test *P* = 0.001), indicating that some groups occupy broader regions of multivariate chemical space than others (Figure 6B). Such dispersion patterns align with the concept of chemical disparity, whereby selection can favor enhanced dissimilarity among related taxa as a route to escape specialist enemies (Becerra, 2007).

### Phylometabolomic congruence with modern systematics

Finally, the tripartite chemical structure recovered here, separating Magnoliids, Monocots, and Eudicots, recapitulates the major angiosperm framework established by molecular phylogenetics. Whereas 20th‐century classifications (e.g., Cronquist, 1981) treated ‘dicots’ as a coherent assemblage, APG‐based systematics formalized the non‐monophyly of that group and recognized Magnoliids as a distinct deep lineage (APG IV, 2016). Recent large‐scale nuclear phylogenomics provides an expanded, independent backbone for angiosperm relationships, reinforcing that this clade‐level partition reflects deep evolutionary history (Zuntini et al., 2024). Our global phylometabolomic analyses independently converge on this same partition: Magnoliids consistently occupy a distinct region of chemical space rather than clustering with Eudicots, consistent with lineage‐specific investment in lipophilic scaffolds and long‐standing alkaloid/lignan biosynthetic routes. Monocots, in turn, emerge as a chemically cohesive lineage with a contrasting physicochemical profile, mirroring their strong molecular monophyly (Zuntini et al., 2024). Together, these patterns indicate that specialized metabolism retains a deep phylogenetic imprint at macroevolutionary scales and that broad clade boundaries are traceable not only in genomes, but also in metabolite repertoires. This congruence supports the use of phylometabolomic data as a complementary axis for interpreting angiosperm evolution and for generating testable predictions about pathway innovation, constraint, and ecological function across lineages.

## CONCLUSION

Collectively, our analyses support a general macroevolutionary regime in which angiosperm chemical diversification is persistently turnover‐dominated, indicating that deep‐lineage disparity arises primarily through recurrent replacement of metabolites rather than directional accumulation. At the same time, this compositional dynamism does not imply unstructured drift: major clades and families occupy distinct physicochemical and elemental neighborhoods, consistent with conserved constraints on molecular design, transport, storage, and biosynthetic investment. Thus, angiosperm specialized metabolism appears to evolve through continuous reinvention of chemical identity within lineage‐specific constraint corridors, linking macroecological dynamics (replacement) to macroevolutionary structure (clade‐resolved chemical architectures). By integrating open chemical occurrence data with standardized taxonomy in a fully reproducible workflow, our framework moves beyond traditional chemotaxonomy to phylometabolomics, providing quantitative benchmarks and a reusable resource for dissecting how ecology and biosynthesis jointly shape plant chemical diversity across deep time.

## MATERIALS AND METHODS

### Data sources and acquisition workflow

All chemical occurrence data were obtained from the LOTUS database (Rutz et al., 2022) (https://lotus.naturalproducts.net/), an open‐access repository that links natural product structures to their biological sources. The complete dataset was accessed locally via MongoDB using the mongolite package in R v4.4.2. Analyses were conducted through a fully scripted, reproducible workflow comprising data ingestion, taxonomic harmonization, and the computation of diversity metrics across 12 representative angiosperm families selected to span three major evolutionary lineages: Magnoliids (Annonaceae, Lauraceae, Magnoliaceae, Piperaceae), Monocots (Amaryllidaceae, Dioscoreaceae, Orchidaceae, Poaceae), and Eudicots (Asteraceae, Bignoniaceae, Fabaceae, Rutaceae).

### Taxonomic and chemical standardization

Plant names were standardized to currently accepted taxa using the World Flora Online backbone (WFO, 2025) (https://www.worldfloraonline.org/). Synonyms, orthographic variants, and unresolved names were programmatically matched to accepted species, genus, and family ranks; records lacking unambiguous taxonomic assignments were excluded. Chemical structures were consolidated at the InChIKey level to guarantee structural uniqueness. Physicochemical descriptors, including molecular weight (MW), topological polar surface area (TPSA), and lipophilicity (XLogP), along with the fraction of sp^3^‐hybridized carbons (Fsp^3^) as a proxy for structural complexity, were retrieved directly from LOTUS metadata. Furthermore, atomic composition counts were extracted to derive the Carbon‐to‐Oxygen (C/O) ratio and total Nitrogen content per metabolite to assess metabolic cost constraints, as well as ClassyFire taxonomic classifications were retrieved directly from LOTUS metadata (Djoumbou Feunang et al., 2016).

### Filtering and construction of the chemical core

To isolate lineage‐specific metabolic traits from ubiquitous housekeeping chemistry, the initial dataset (159 192 occurrences) was subjected to a two‐step filtration protocol. First, a promiscuity filter excluded compounds recorded in more than six families or present in ≥20% of all species globally. Second, a within‐family support filter removed singleton records (compounds found in only one species per family) to minimize sampling artifacts. The resulting ‘chemical core’ (77 404 occurrences) was used to construct binary species × compound incidence matrices for downstream analyses. All downstream analyses reported here were performed on the resulting chemical core, ensuring that conclusions are not driven by globally promiscuous metabolites or within‐family singletons.

### Chemotaxonomic profiling and clustering

Chemotaxonomic structure was evaluated using hierarchical clustering of biosynthetic profiles. To generate the global dendrogram and biosynthetic heatmap, chemical‐class abundance matrices were Hellinger‐transformed to reduce the weight of dominant classes, converted to *Z*‐scores, and clustered using Ward's method (ward.D2) based on Euclidean distances. The congruence between chemical clustering and APG IV phylogeny was visualized using the ComplexHeatmap and dendextend packages. The robustness of the clustering topology was assessed via multiscale bootstrap resampling (*n* = 1000) using the pvclust package.

### Chemical diversity and rarefaction

Family level chemical diversity was quantified using Hill numbers (*q* = 0, 1, 2), representing richness, Shannon‐effective diversity, and Simpson‐effective diversity (Chao et al., 2014; Petrén et al., 2024). To account for uneven sampling effort while retaining rare compounds, diversity estimates were standardized using coverage‐based rarefaction and extrapolation (iNEXT package) to a target coverage of 0.95, rather than rarefying to a fixed sample size.

### Multivariate gradients and β‐diversity partitioning

To ensure comparability in dissimilarity metrics across lineages with disparate species counts, multivariate analyses were conducted on a standardized subsample of 200 species per family. This threshold was validated through a robust rarefaction simulation (*N* = 20 to *N* = 1000), which confirmed parameter stability for dispersion estimates at this sampling depth (Figure S1).

Using this standardized dataset, global chemical space was mapped via principal coordinate analysis (PCoA) based on Jaccard dissimilarity matrices. Differences in chemical composition centroids were tested using PERMANOVA (999 permutations), and within‐family heterogeneity was evaluated using multivariate dispersion (betadisper). Total β‐diversity was further decomposed into turnover (β_SIM) and nestedness (β_SNE) components following the framework of Baselga (2010), computed with the betapart package to determine whether chemical differentiation is driven by compound replacement or subset retention.

### Machine learning, network topology, and phylogenetic signal

To identify diagnostic chemical markers for each lineage, a robust Random Forest classifier (ranger package) was trained on species‐level chemical profiles to predict APG clades, utilizing 2000 trees; variable importance was assessed via Mean Decrease Accuracy (MDA). Bipartite network analysis was conducted using the bipartite package; the family–class incidence matrix was log‐transformed (log1p) to mitigate the dominance of ubiquitous classes before computing quantitative modularity (*Q*) and the *H*_2_′ specialization index. Furthermore, to test for evolutionary conservation in biosynthetic traits (e.g., plasticity and richness), we calculated Blomberg's *K* statistic using the phytools package, utilizing a phenetic tree (UPGMA) built from chemical distances to summarize biosynthetic affinities.

### Univariate statistical analysis and environment

For univariate physicochemical and elemental traits (MW, XLogP, Fsp^3^, Nitrogen, C/O), statistical significance was determined using Kruskal–Wallis tests followed by pairwise Wilcoxon rank‐sum tests with Benjamini–Hochberg (FDR) correction for multiple comparisons (multcompView). Key packages included dplyr and tidyr for data manipulation; ggplot2, ggalluvial, and circlize for visualization; and ape for phylogenetic tree handling.

## CONFLICT OF INTEREST

The authors declare no conflicts of interest.

## Supporting information


**Figure S1.** Subsampling stability of within‐lineage chemical dispersion estimates.
**Figure S2.** Family‐wide metabolic class composition mapped onto the angiosperm backbone.
**Figure S3.** Family‐specific occupation of physicochemical space by major chemical classes.

**Table S1.** Global prevalence of excluded promiscuous metabolites in LOTUS.

## Data Availability

All data supporting the findings of this study, including curated occurrence tables, processed matrices used for all analyses, and the reproducible analysis workflow (scripts and configuration files), are available in a public repository at https://github.com/carloscarollo‐UFMS/phylometabolomics. The repository contains instructions to reproduce all figures and tables presented in the manuscript. Additional supporting data and materials are provided as Supporting Information.
