# Supplementary material for: Open data phylometabolomics reveals turnover‐dominated chemical divergence and clade‐specific physicochemical regimes across angiosperms
Source: Plant J. 2026 Mar 23;125(6):e70820. doi: 10.1111/tpj.70820 (PMC13008826; doi:10.1111/tpj.70820)
Supplement: Supplementary file 1 — Figure S1. Subsampling stability of within‐lineage chemical dispersion estimates. Figure S2. Family‐wide metabolic class composition mapped onto the angiosperm backbone. Figure S3. Family‐specific occupation of physicochemical space by major chemical classes. [file TPJ-125-0-s001.docx]

**Table S1.** Global prevalence of excluded promiscuous metabolites in LOTUS.

List of the 353 metabolites excluded by the promiscuity filter, reported by InChIKey. For each compound, we provide its occurrence breadth within the focal dataset (n_families, n_species) and its prevalence across the full LOTUS resource (n_families_global, n_species_global). prop_species_global indicates the proportion of species represented by the compound under the global reference used in the analysis. This table supports the summary statistics reported in the main text (mean ± SD and observed maxima for global family and species coverage).


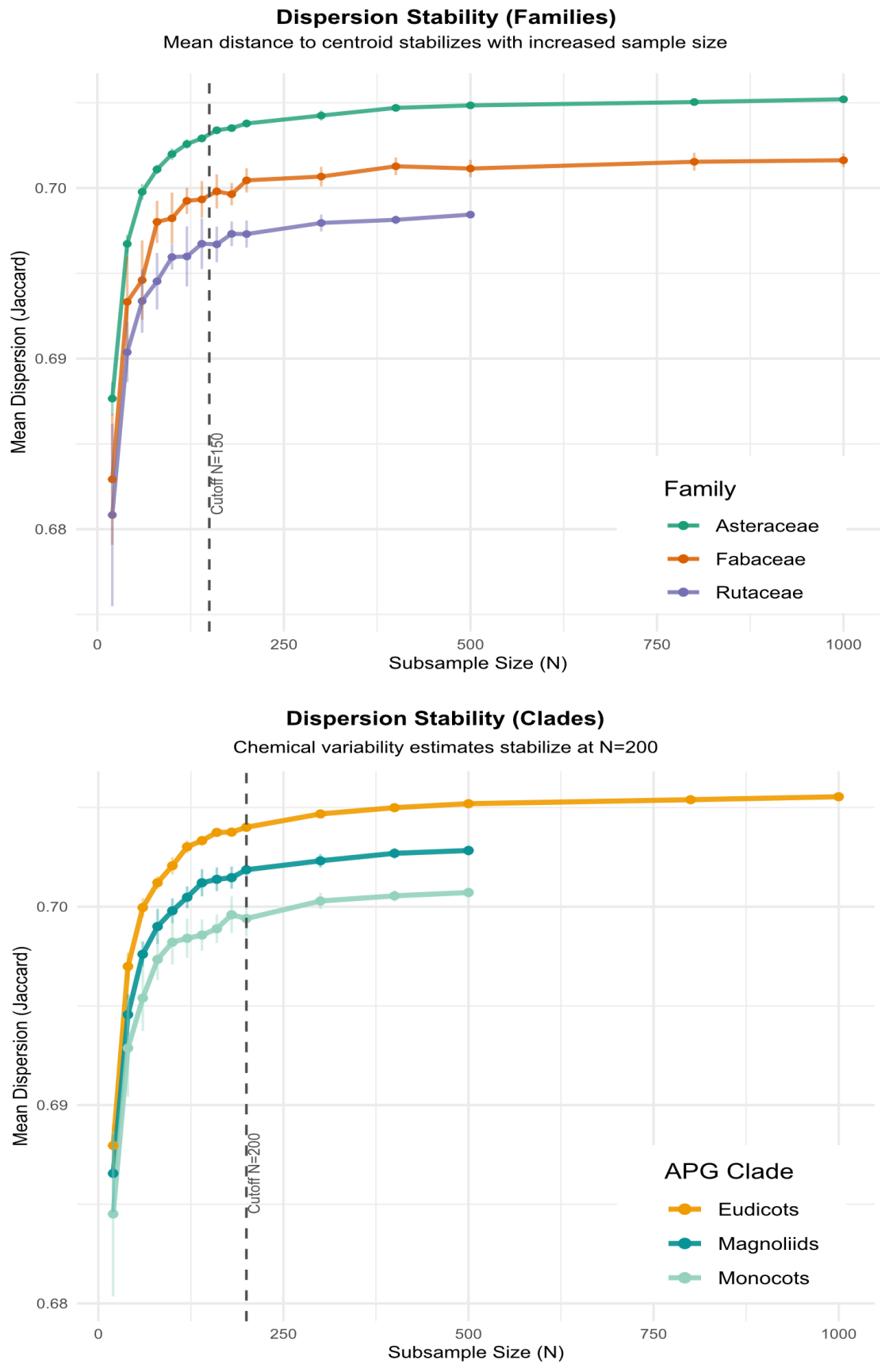


**Figure S1.** Subsampling stability of within-lineage chemical dispersion estimates.

(A) Families with sufficient sampling depth (≥300 species). (B) Major APG clades with sufficient sampling depth (≥300 species). For each group, we repeatedly subsampled k species without replacement (k = 20–200 in steps of 20, then 300, 400, 500, 800, 1000; 10 replicates per k), built a binary species × InChIKey incidence matrix, computed Jaccard dissimilarities (vegdist, binary = TRUE), and estimated multivariate dispersion as the mean distance to the centroid (betadisper). Points show the mean dispersion across replicates and error bars indicate ±1 SD. The dashed vertical line marks N = 200, the standardized cutoff used in the main analyses, showing that dispersion estimates largely stabilize by this sample size.


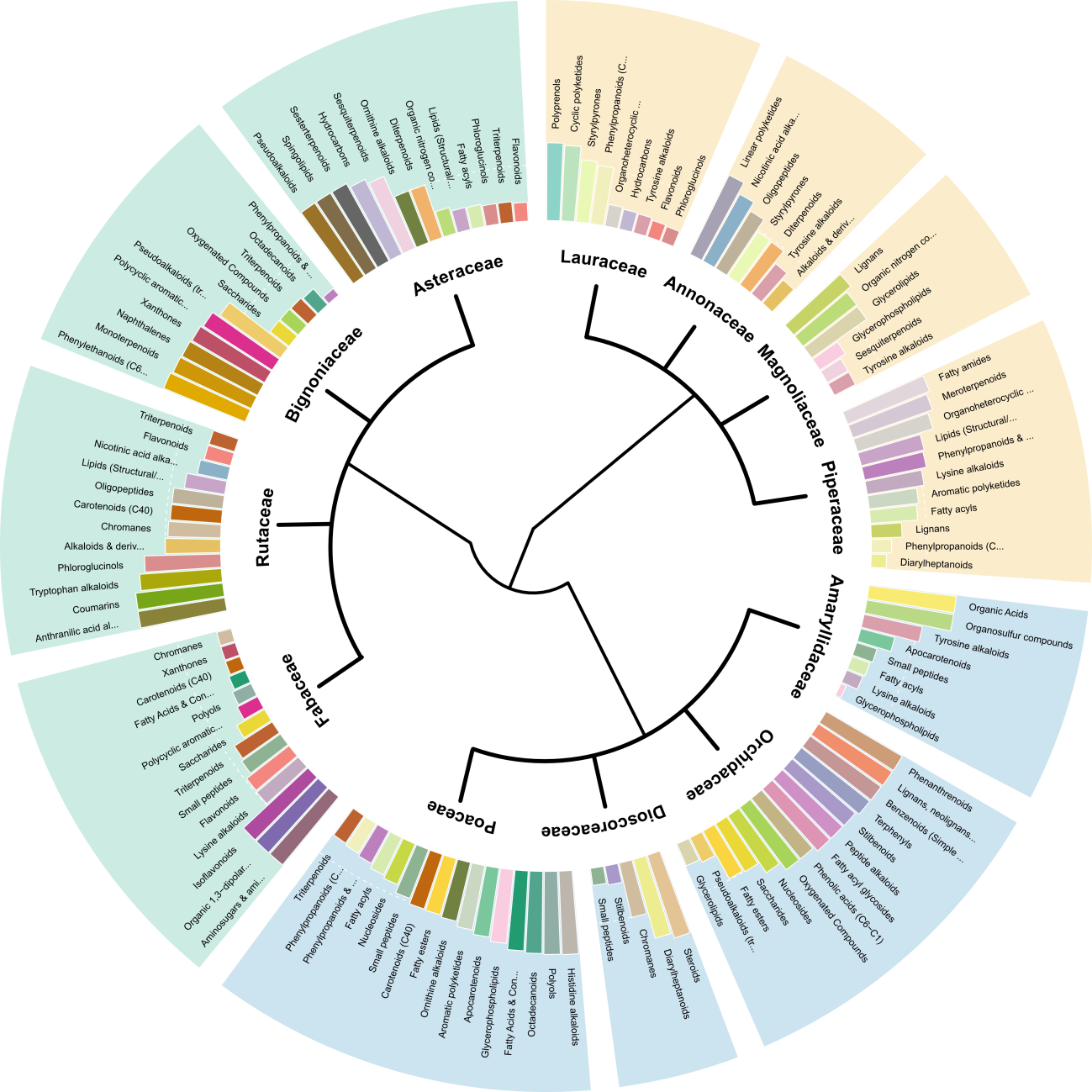


**Figure S2.** Family-wide metabolic class composition mapped onto the angiosperm backbone.

Circular phylogram summarizing the relative investment of each focal family in major specialized-metabolite classes. For each family, the outer stacked bars show the proportional contribution (%) of chemical classes (from the curated LOTUS-derived occurrence dataset; InChIKey-level occurrences) to the family’s overall chemical profile, with labels indicating the dominant classes per lineage. Background wedges group families by APG clade (Magnoliids, Monocots, Eudicots), highlighting how clade-level chemical “footprints” emerge from distinct, family-specific biosynthetic allocations.


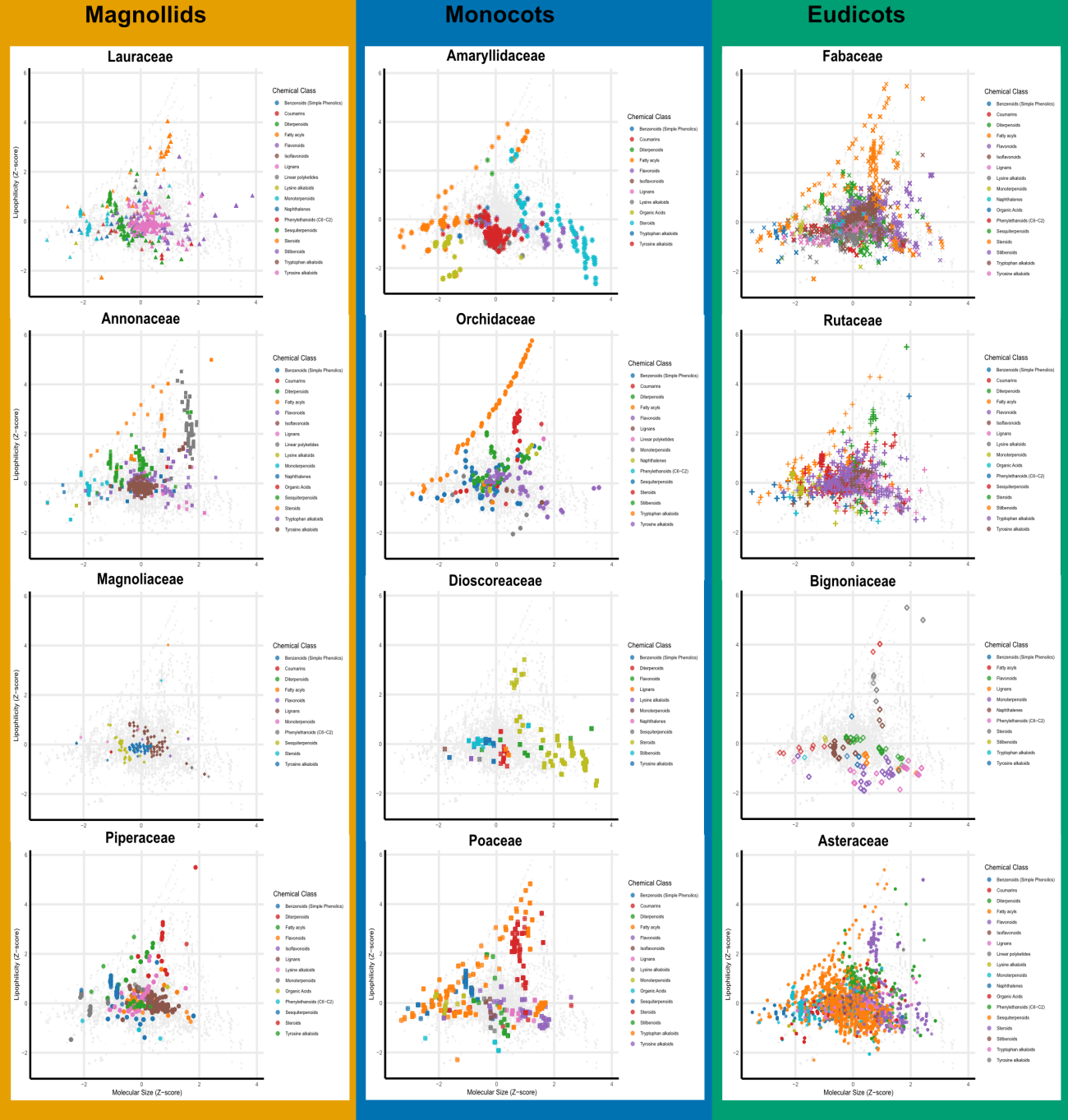


**Figure S3.** Family-specific occupation of physicochemical space by major chemical classes.

Scatterplots show the distribution of metabolites within each of the 12 focal families across two standardized physicochemical axes: molecular size (x-axis; Z-score) and lipophilicity (y-axis; Z-score). Points are colored by chemical class (see legend), and the light gray background cloud shows the full angiosperm-wide reference distribution used for comparison. Panels are grouped by APG clade (Magnoliids, Monocots, Eudicots), illustrating how each clade-level footprint is assembled from family-specific class mixtures and distinct physicochemical niches.
